# Supplementary material for: Multiple Environmental Signaling Pathways Control the Differentiation of RORγt-Expressing Regulatory T Cells
Source: Front Immunol. 2020 Jan 8;10:3007. doi: 10.3389/fimmu.2019.03007 (PMC6961548; doi:10.3389/fimmu.2019.03007)
Supplement: Supplementary file 1 [file Data_Sheet_1.PDF]

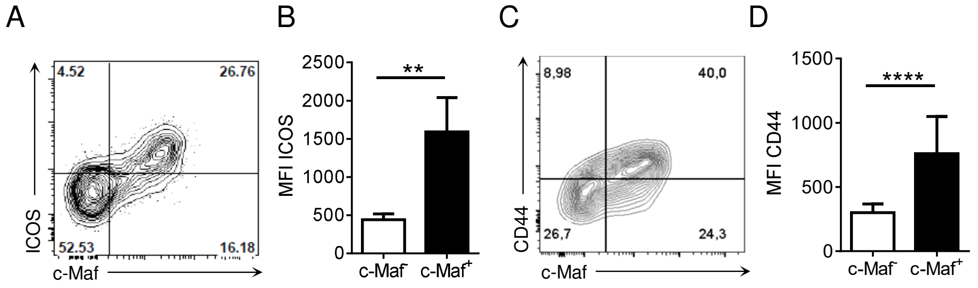

Figure S1. **c-Maf+ Tregs have an activated phenotype.** (A, C) Representative flow cytometry expression profiles of ICOS (A) or CD44 (C) versus c-Maf among Treg cells in mLN of WT mice (gate $CD4^+$ $Foxp3^+$). (B, D) Histograms show the MFI of ICOS (B) and CD44 (D) among c-Maf- and c-Maf+ Treg cells. Histograms represent the mean ± SD of at least five individual mice. Difference between groups is determined by a Mann–Whitney test for two-tailed data. **$p < 0.01$; ****$p < 0.0001$
