# Supplementary material for: Multiple Environmental Signaling Pathways Control the Differentiation of RORγt-Expressing Regulatory T Cells
Source: Front Immunol. 2020 Jan 8;10:3007. doi: 10.3389/fimmu.2019.03007 (PMC6961548; doi:10.3389/fimmu.2019.03007)
Supplement: Supplementary file 2 [file Data_Sheet_2.PDF]

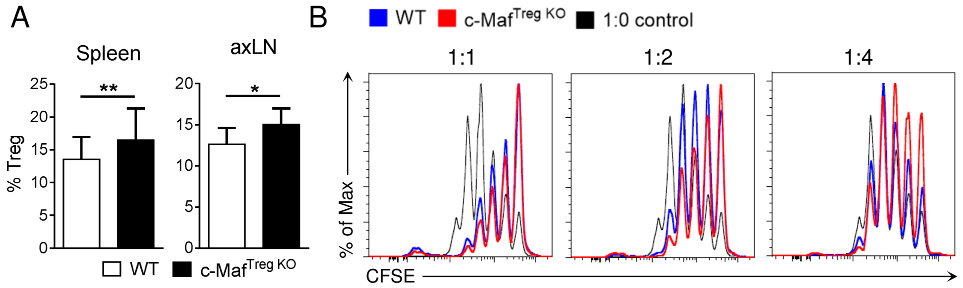

Figure S2. **c-Maf-deficient Tregs have a suppressive phenotype.** (A) Frequency of Treg cells in the spleen or axillary lymph nodes of WT and c-Maf$^{Treg\ KO}$ mice (gate CD4^{+}). (B) Histograms showing CFSE staining profiles of conventional CD4 T cells in a Treg suppression assay *in vitro*. Results are representative of at least three independent experiments; histograms represent the mean ± SD of five individual mice. Difference between groups is determined by a Mann–Whitney test for two-tailed data. *p < 0.05; **p < 0.01
