# Supplementary material for: Multiple Environmental Signaling Pathways Control the Differentiation of RORγt-Expressing Regulatory T Cells
Source: Front Immunol. 2020 Jan 8;10:3007. doi: 10.3389/fimmu.2019.03007 (PMC6961548; doi:10.3389/fimmu.2019.03007)
Supplement: Supplementary file 4 [file Data_Sheet_4.PDF]

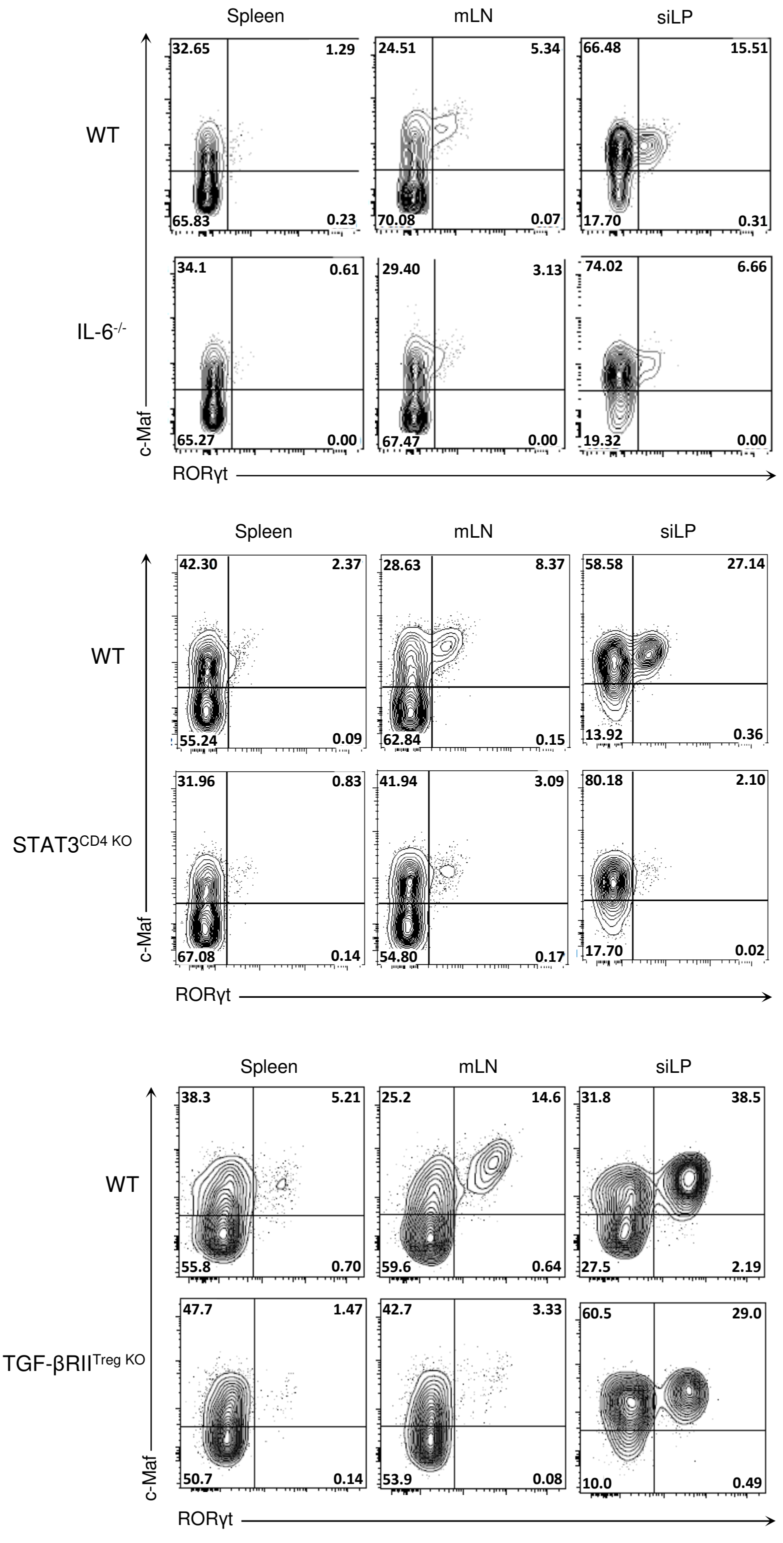

Figure S4. **IL-6/STAT3 and TGF-β signaling promote RORγt expression in Tregs independently of c-Maf**. Representative flow cytometry expression profiles of c-Maf versus RORγt in Treg cells in the indicated organs of the indicated mice strains (gate $CD4^+$ $Foxp3^+$). Results are representative of at least three independent experiments.
