# Supplementary material for: Multiple Environmental Signaling Pathways Control the Differentiation of RORγt-Expressing Regulatory T Cells
Source: Front Immunol. 2020 Jan 8;10:3007. doi: 10.3389/fimmu.2019.03007 (PMC6961548; doi:10.3389/fimmu.2019.03007)
Supplement: Supplementary file 5 [file Data_Sheet_5.PDF]

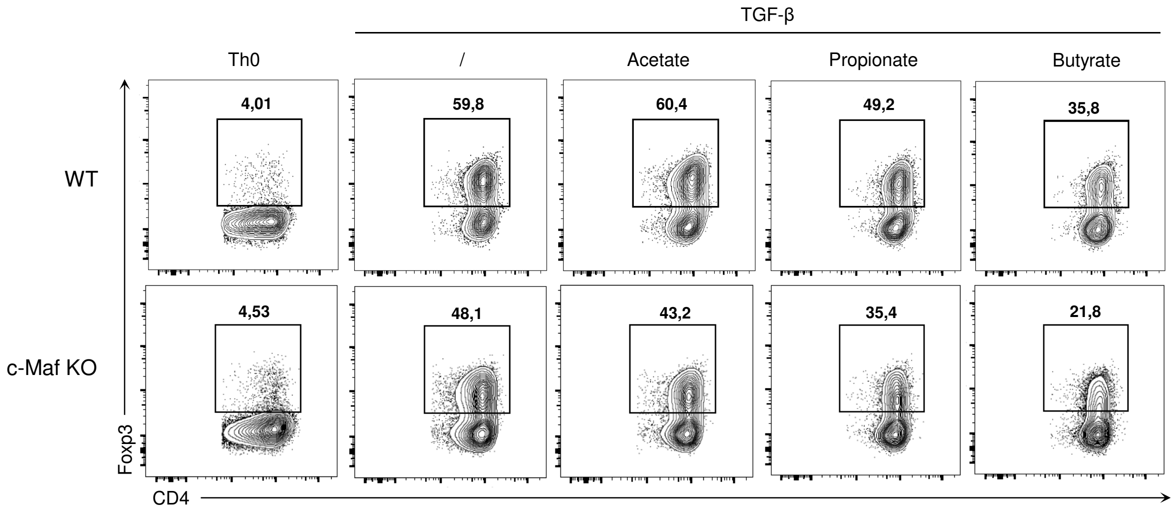

Figure S5. **In vitro Treg differentiation in presence of short-chain fatty acids.** Naïve WT or c-Maf-deficient CD4 T cells were activated *in vitro* for 72h in presence of TGF-β and small chain fatty acids. Representative flow cytometry expression profiles of Foxp3 in CD4 cells in the indicated conditions; gating strategy of Fig. 4E, F. Results are representative of at least three independent experiments.
