# Supplementary material for: Multiple Environmental Signaling Pathways Control the Differentiation of RORγt-Expressing Regulatory T Cells
Source: Front Immunol. 2020 Jan 8;10:3007. doi: 10.3389/fimmu.2019.03007 (PMC6961548; doi:10.3389/fimmu.2019.03007)
Supplement: Supplementary file 6 [file Data_Sheet_6.PDF]

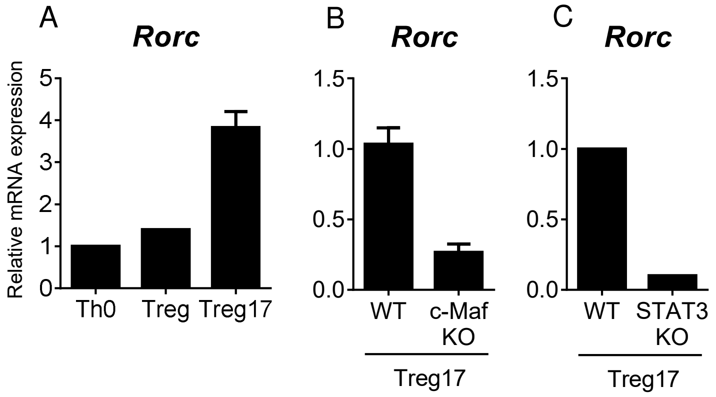

Figure S6. **Transcription control of RORγt expression in Tregs.** Naïve CD4 T cells from Foxp3-Cre-YFP (A, B), c-Maf$^{Treg\ KO}$ (B), or WT and STAT3 KO (C) mice were activated *in vitro* in presence of polarizing cytokines for 72h (Treg: TGF-β, IL-2; Treg17: TGF-β, IL-2, IL-6). Treg cells from A and B were further purified by FACS based on YFP expression. Histograms show mRNA expression of *Rorc* relative to RPL32, with Th0 (A) or WT Treg17 (B, C) set to 1. Results are representative of at least three independent experiments; histograms represent the mean ± SD of 2-3 individual samples.
