# Supplementary material for: Multiple Environmental Signaling Pathways Control the Differentiation of RORγt-Expressing Regulatory T Cells
Source: Front Immunol. 2020 Jan 8;10:3007. doi: 10.3389/fimmu.2019.03007 (PMC6961548; doi:10.3389/fimmu.2019.03007)
Supplement: Supplementary file 7 [file Data_Sheet_7.PDF]

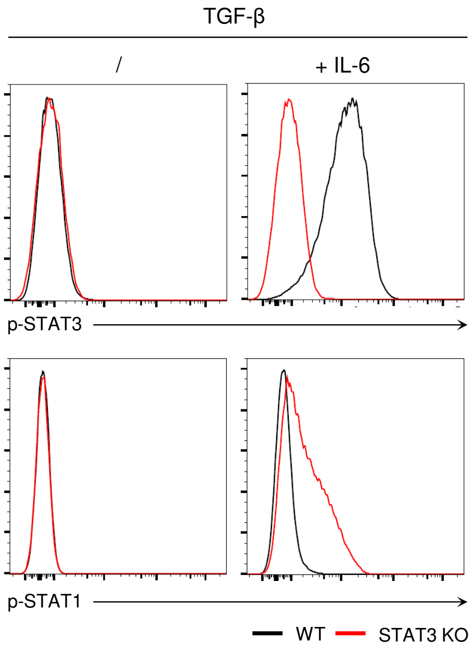

Figure S7. **In absence of STAT3 in Tregs, IL-6 signals via STAT1.** Histograms show the expression of pSTAT3 and pSTAT1 among Treg cells polarized *in vitro* in presence of TGF-β, with or without IL-6 (gate $CD4^+$).
