# Supplementary material for: Multiple Environmental Signaling Pathways Control the Differentiation of RORγt-Expressing Regulatory T Cells
Source: Front Immunol. 2020 Jan 8;10:3007. doi: 10.3389/fimmu.2019.03007 (PMC6961548; doi:10.3389/fimmu.2019.03007)
Supplement: Supplementary file 8 [file Data_Sheet_8.PDF]

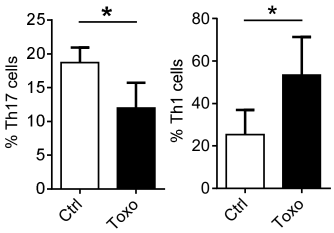

Figure S8. **Infection with *Toxoplasma gondii* is characterized by a Th1 inflammatory environment.** Histograms show the frequency of Th17 cells and Th1 cells in siLP of mice after *Toxoplasma gondii* infection. Histograms represent the mean ± SD of five individual mice. Difference between groups is determined by a Mann–Whitney test for two-tailed data. $^{**}p < 0.01$; $^{****}p < 0.0001$
