# Supplementary material for: Multiple Environmental Signaling Pathways Control the Differentiation of RORγt-Expressing Regulatory T Cells
Source: Front Immunol. 2020 Jan 8;10:3007. doi: 10.3389/fimmu.2019.03007 (PMC6961548; doi:10.3389/fimmu.2019.03007)
Supplement: Supplementary file 9 [file Data_Sheet_9.PDF]

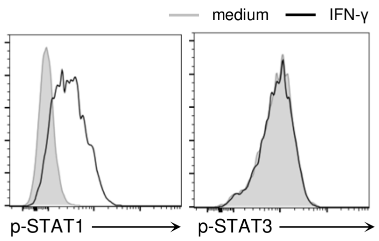

Figure S9. **IFN-γ does not impede IL-6-mediated STAT3 phosphorylation in iTregs.** Histograms show the expression of pSTAT1 and pSTAT3 among Treg cells polarized *in vitro* in presence of TGF-β, IL-2 and IL-6 with or without IFN-γ (10 ng/mL, gate $CD4^+$ $Foxp3^+$).
