# Supplementary material for: Multiple Environmental Signaling Pathways Control the Differentiation of RORγt-Expressing Regulatory T Cells
Source: Front Immunol. 2020 Jan 8;10:3007. doi: 10.3389/fimmu.2019.03007 (PMC6961548; doi:10.3389/fimmu.2019.03007)
Supplement: Supplementary file 10 [file Data_Sheet_10.PDF]

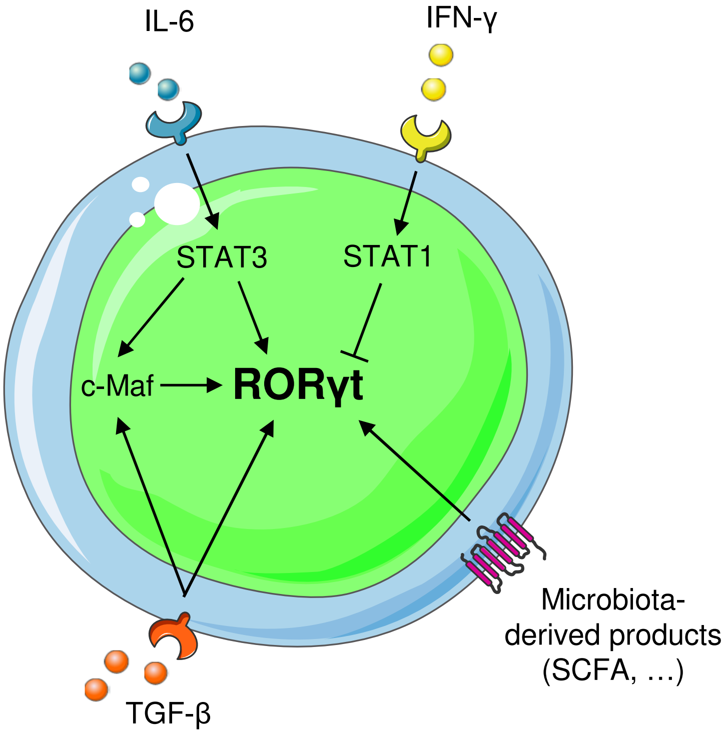

Figure S10. **Model of RORγt induction in Tregs.** The differentiation of specialized cell subsets is a balancing act: multiple positive and negative signals are integrated in order to tailor cell specialization to the immune context. Signals derived from a complex microbiota, or IL-6/STAT3 and TGF-β signaling induce RORγt expression in Tregs in a c-Maf-dependent or independent fashion. Contrastingly, inflammatory IFN-γ/STAT1 signaling opposes RORγt expression in Tregs in a c-Maf independent fashion.
